# Suppressor of Cytokine Signaling 4 (SOCS4) Protects against Severe Cytokine Storm and Enhances Viral Clearance during Influenza Infection

**DOI:** 10.1371/journal.ppat.1004134

**Published:** 2014-05-08

**Authors:** Lukasz Kedzierski, Edmond M. Linossi, Tatiana B. Kolesnik, E. Bridie Day, Nicola L. Bird, Benjamin T. Kile, Gabrielle T. Belz, Donald Metcalf, Nicos A. Nicola, Katherine Kedzierska, Sandra E. Nicholson

**Affiliations:** 1 Walter and Eliza Hall Institute of Medical Research, Parkville, Melbourne, Victoria Australia; 2 Department of Medical Biology, The University of Melbourne, Parkville, Melbourne, Victoria, Australia; 3 Department of Microbiology and Immunology, The University of Melbourne, Parkville, Melbourne, Victoria, Australia; National Institutes of Health, United States of America

## Abstract

Suppressor of cytokine signaling (SOCS) proteins are key regulators of innate and adaptive immunity. There is no described biological role for SOCS4, despite broad expression in the hematopoietic system. We demonstrate that mice lacking functional SOCS4 protein rapidly succumb to infection with a pathogenic H1N1 influenza virus (PR8) and are hypersusceptible to infection with the less virulent H3N2 (X31) strain. In SOCS4-deficient animals, this led to substantially greater weight loss, dysregulated pro-inflammatory cytokine and chemokine production in the lungs and delayed viral clearance. This was associated with impaired trafficking of influenza-specific CD8 T cells to the site of infection and linked to defects in T cell receptor activation. These results demonstrate that SOCS4 is a critical regulator of anti-viral immunity.

## Author Summary

The suppressor of cytokine signaling proteins are key regulators of immunity. As yet there is no described biological role for SOCS4, despite its broad expression in cells of the immune system. Given the important role of other SOCS proteins in controlling the immune response, we have generated SOCS4-mutant mice and used a mouse influenza infection model to investigate the biological function of SOCS4. We demonstrate that mice lacking SOCS4 rapidly succumb to infection with a pathogenic H1N1 influenza virus and are hypersusceptible to infection with the less virulent H3N2 strain. This is the first demonstration of a functional phenotype in SOCS4-deficient mice. Our study reveals that in SOCS4-deficient animals, there is a dysregulated pro-inflammatory cytokine and chemokine production in the lungs and delayed viral clearance. This is associated with impaired trafficking of virus-specific CD8 T cells to the site of infection and linked to defects in T cell receptor activation. These results demonstrate that SOCS4 is a critical regulator of anti-viral immunity. Understanding the regulation of the inflammatory response to influenza is particularly relevant given the current climate concerning pandemic influenza outbreaks.

## Introduction

Influenza is a highly infectious, acute respiratory disease that causes profound morbidity and mortality. Annual seasonal influenza epidemics result in ∼500,000 deaths worldwide and substantial losses to global economies [1]. The development of a “cytokine storm” coupled with damage to pulmonary epithelium has been consistently observed in severe cases of influenza infection in humans. The mechanisms underlying this pathology, and an understanding of why some individuals respond excessively to virus, to an extent that results in hospitalisation or death, remains relatively unexplored.

The initial innate immune response to viral infection is characterized by an influx of neutrophils, monocytes and macrophages into the lung parenchyma and alveolar spaces, with the elevated levels of inflammatory cytokines/chemokines correlating strongly with pathogenesis and viral load [2]. However, exaggerated cytokine and chemokine responses have been observed in the lungs of critically ill patients in the absence of high viral load [3], suggesting that inflammation-driven pathology can occur independently of viral load.

The adaptive response subsequently results in the generation of strain-specific B cells and cross-strain protective CD4 and CD8 T cells. Influenza-specific CD8 T cells are largely responsible for host immunity to primary influenza infection and operate to promote the efficient elimination of virus, and host recovery, via the production of pro-inflammatory cytokines and direct killing of virus-infected cells [4]. Acquisition of these effector functions occurs in the draining lymph nodes where upon T cell receptor (TCR) recognition of the influenza-specific peptide:MHC complex, CD8 T cells become activated and then migrate to the infected lungs.

Cytokine binding to the cognate receptor complexes triggers an intracellular signaling cascade, most often coupled to the JAK-STAT pathway, which orchestrates an intricate series of transcriptional changes leading to the appropriate cellular response. The suppressors of cytokine signaling (SOCS) proteins are key negative regulators of the JAK/STAT pathway and are thus involved in the fine-tuning of the cytokine networks responsible for an adequate and efficient innate and adaptive immune response [5]. The family is composed of eight members, SOCS1 to 7 and cytokine-inducible Src-homology 2 protein (CIS) [6]. All proteins share a central SH2 domain and carboxyl-terminal SOCS box, but differ in their amino termini. SOCS4 to 7 are particularly distinguished by a long N-terminal region, which bears little homology to other SOCS proteins [7]. The SOCS box interacts with elongins B and C, and together with Rbx2 and Cullin-5 forms an E3 ubiquitin ligase [8]. The SOCS proteins therefore act as adaptors to target substrates bound to their SH2 or N-terminal regions for ubiquitination and proteasomal degradation [9]. In addition, SOCS1 and 3 can bind directly to JAK via their kinase inhibitory region (KIR) and SH2 domains, inhibiting JAK phosphorylation of substrates and downstream signaling [10], [11]. CIS and SOCS2 are thought to bind to phosphotyrosine residues within the receptor cytoplasmic domains to block recruitment of other signalling intermediates [12], [13], [14].

The generation of knockout mice has proven a powerful tool in defining the physiological role of the SOCS proteins. SOCS1 for instance, was revealed as a critical regulator of IFNγ signaling and γ_c_-cytokine-dependent T cell homeostasis [15], SOCS2 as a regulator of growth hormone signaling [16] and conditional deletion of the *Socs3* gene has identified a role for SOCS3 in regulating IL-6 and G-CSF signaling [17], [18]. Although a wealth of information is available on the role of CIS and SOCS1-3, there is much less data regarding the targets and pathways regulated by the remaining family members, including SOCS4.

*In vitro* studies have suggested that SOCS4 is involved in regulating epidermal growth factor (EGF) signaling [19], and indeed the SOCS4-SH2 domain binds with high affinity to a phosphopeptide corresponding to an EGF receptor (EGFR) autophosphorylation site (Tyr1092; 0.5 µM) [20]. This later study provides some information as to the binding preferences of the SOCS4-SH2 domain [20], however the relevance of the interaction with the EGFR remains to be elucidated. Other studies have suggested that SOCS4 may be regulated by parasitic infection [21], is linked to better outcomes in cancer patients [22], or may regulate pre-granulosa cells during folliculogenesis [23]. Thus far, none of these studies present a compelling case for a physiological role for SOCS4.

In the current study, we have generated the first loss of function allele of murine *Socs4*, a point mutation identified in a library of ENU-mutagenised mice [24]. At the protein level, this mutation is predicted to cause the substitution of a stop codon for arginine 108, resulting in deletion of the remaining 90 residues of the N-terminal domain, as well as the entire SH2 domain and SOCS box. Given that SOCS proteins play a critical role in regulating immune responses, and expression of SOCS4 in lymphocytes, we investigated the role of SOCS4 in a defined viral infection model in which T cells regulate pathogen clearance. We found that *Socs4^R108X/R108X^* mice were highly susceptible to infection with influenza A, showing increased morbidity and a delay in viral clearance comparable to that observed for mice lacking CD8 T cells [25] or IL-18 [26]. The increased lethality appeared to result from an elevation in key pro-inflammatory cytokines and chemokines, such as IL-6, IFNγ and MCP-1; whilst in the latter phase of the infection, *Socs4^R108X/R108X^* mice displayed impaired trafficking of virus-specific CD8 T cells to the lungs. The defect in trafficking appeared to be qualitatively linked to the activation status of these cytotoxic T lymphocytes and reveals a novel role for SOCS4 as a positive regulator of TCR signaling.

## Results

### SOCS4 protects mice against virulent influenza infection

**Figure 1:**
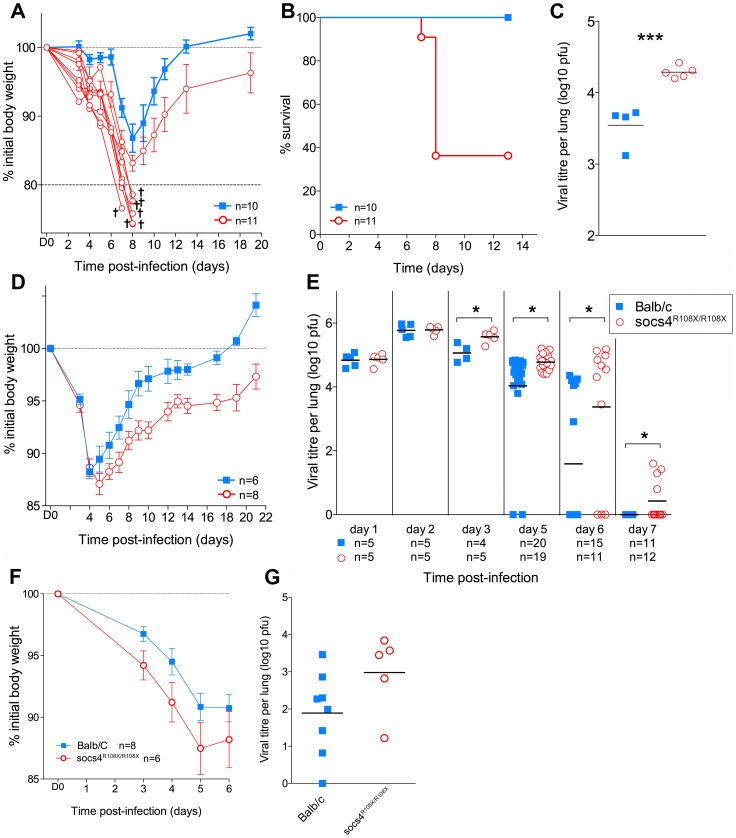
*Socs4^R108X/R108X^* mice show increased susceptibility to influenza A infection. (A) Mice were infected i.n. with 20 pfu H1N1 PR8 virus and weight loss monitored for 20 days. Mice that lost more than 20% of their initial body weight (shown as separate lines in the graph) were considered moribund and removed from the study. (B) Kaplan-Meier survival plot of *Socs4^R108X/R108X^* and Balb/c control mice. Survival of *Socs4^R108X/R108X^* mice was significantly lower than controls (p = 0.002, Log-rank test). Combined data from two independent experiments are shown. (C) Comparison of lung viral titres on day 5 post-PR8 infection, *** indicates p value of 0.008 (t-test). (D) *Socs4^R108X/R108X^* mice showed significantly greater weight loss than Balb/c controls (p = 0.005) and slower recovery following infection with 10^3^pfu of H3N2 X31 influenza virus. Data shown are representative of independent experiments (n = 4). (E) *Socs4^R108X/R108X^* mice display significantly higher lung viral titres than control mice following infection with 10^3^pfu of H3N2 X31 influenza virus. Combined data from independent experiments are shown, * indicates p value <0.05. (F–G) Wild-type mice reconstituted with *Socs4^R108X/R108X^* bone marrow show increased disease severity following influenza infection. Lethally irradiated wild-type Thy1.1 mice were reconstituted with *Socs4^R108X/R108X^* Thy1.2 or WT Thy1.2 bone marrow and infected with H3N2 X31 influenza A virus. Weight loss (F) was monitored for 6 days, at which time point lungs were harvested for viral titer estimation (G). Pooled data from two independent experiments are shown.

*Socs4^R108X/R108X^* mice were viable, fertile and showed no overt phenotype under steady-state conditions, including normal thymocyte development and composition of peripheral immune cells (Fig. S1). To investigate the role of SOCS4 in the response to viral challenge, homozygous *Socs4^R108X/R108X^* mice and age-matched littermates or Balb/c controls were inoculated intranasally (i.n.) with 20 pfu of the virulent H1N1 influenza strain A/Puerto Rico/8/34 (PR8) and monitored for weight loss. In accordance with ethical guidelines, mice were considered moribund upon losing greater than 20% of their initial body weight, and removed from the study. At this relatively low challenge dose, *Socs4^R108X/R108X^* mice exhibited significantly enhanced disease progression and mortality compared to wild-type controls (Fig. 1A and B) and this correlated with an increased viral load in the lungs (Fig. 1C).

To dissect the defective response in more detail, *Socs4^R108X/R108X^* mice and wild-type controls were subsequently infected i.n. with 10^3^ pfu of the less virulent H3N2 A/Hong Kong x31 (X31) influenza virus. Similarly to infection with PR8, *Socs4^R108X/R108X^* mice showed enhanced susceptibility to X31 infection with significantly greater weight loss (*P* = 0.005; Fig. 1D) and higher lung viral loads (Fig. 1E) when compared to controls. Initially (day 1 and 2 post-infection) there were no differences in viral titers between the groups, indicating that viral uptake and replication are not affected by SOCS4 deficiency. However, *Socs4^R108X/R108X^* mice displayed higher viral titers (0.5 to 1.8 log higher) on day 3 and day 5 post-infection and this difference was greatly exacerbated by day 6 (2 log difference), when the majority of control mice had cleared the infection. Virus was undetectable in the lungs of wild-type controls on day 7, however some of the *Socs4^R108X/R108X^* mice still retained low viral loads (Fig. 1E). The augmented susceptibility to influenza infection was independent of genetic background, with similar results observed in congenic *Socs4^R108X/R108X^* mice on the C3H/He and C57BL/6 backgrounds (data not shown).

To investigate whether the increased susceptibility to influenza derives from a defect in the hematopoietic compartment, we generated chimeric mice by transplanting bone marrow cells from Thy1.2 *Socs4^R108X/R108X^* or Thy1.2 wild-type mice into lethally irradiated Thy1.1 wild-type recipients. The chimeric mice were subsequently infected i.n. with 10^3^ pfu of X31 virus and disease progression monitored for 6 days. Although not significantly different, weight loss and particularly viral load were both increased in *Socs4^R108X/R108X^* mice compared to controls (Fig. 1F and G), indicating that a defect in the hematopoietic compartment most likely contributes to these aspects of the phenotype.

### Morbidity in *Socs4^R108X/R108X^* mice correlates with elevated proinflammatory cytokines

**Figure 2:**
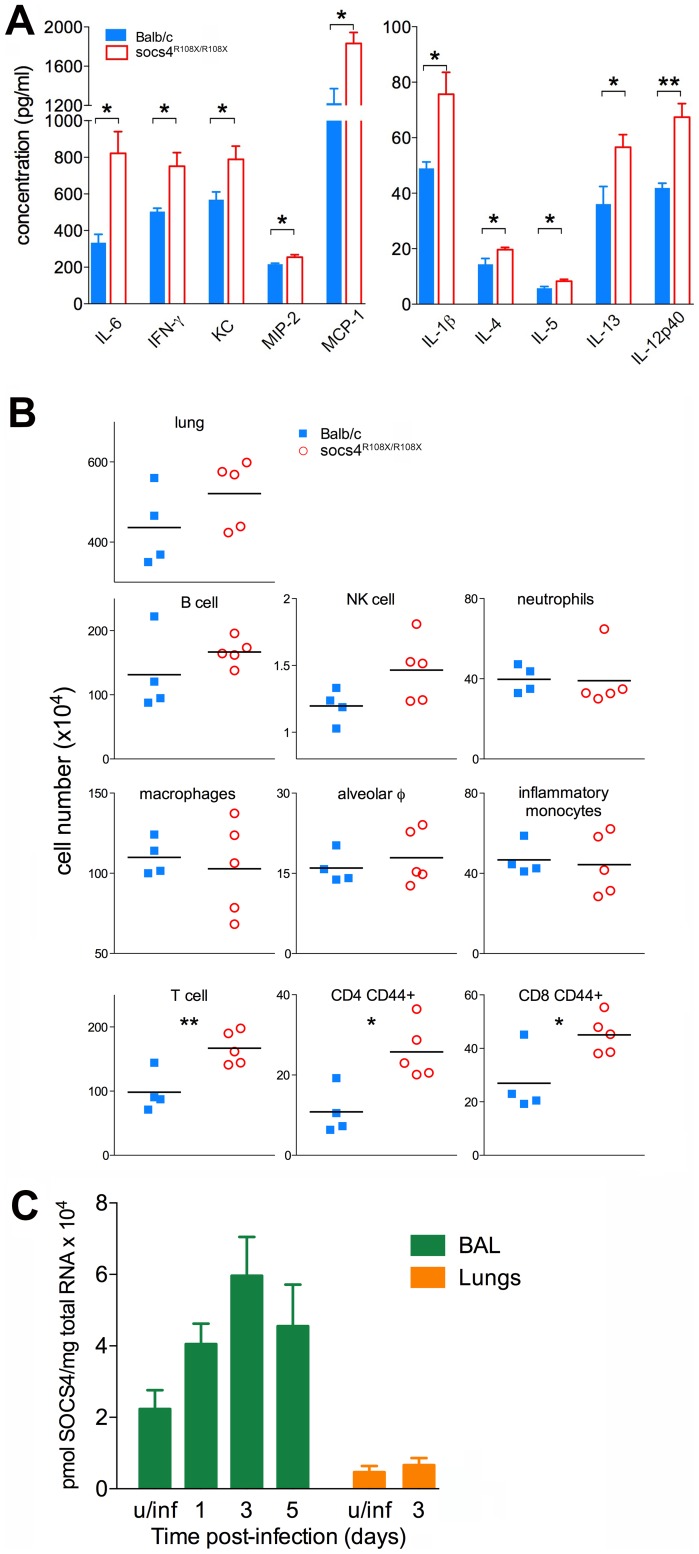
Elevated cytokine and chemokine production in lungs of *Socs4^R108X/R108X^* is associated with an increased influx of T cells. (A) Cytokine and chemokine levels were analysed by ELISA and Bioplex in lung homogenates at day 3 post-infection with X31 virus. Mean data ± S.E.M. are shown for biological replicates (n = 4 for Balb/c, n = 5 for *Socs4^R108X/R108X^*), * indicates p<0.05, **<0.005. (B) Phenotypic analysis of lung hematopoietic subsets in *Socs4^R108X/R108X^* and Balb/c mice at day 3 post-infection. Flow cytometry analysis was performed on homogenized lungs and extracted BAL. Data plotted include combined cell numbers from lungs and BAL, * indicates p<0.05, **<0.005. (C) Expression of *Socs4* mRNA in immune cells recovered from BAL and in the lungs of Balb/c mice infected with X31 virus. Mean data ± S.E.M. are shown for n = 3 biological replicates, u/inf = uninfected.

The poorer outcomes associated with virulent influenza strains are thought to be due to excessive production of proinflammatory cytokines and chemokines. This dysregulated immune response and the resulting lung inflammation and damage is one mechanism by which pandemic infections cause high mortality [27], [28]. Since *Socs4^R108X/R108X^* mice showed greater susceptibility to infection, we compared cytokine and chemokine profiles in lung homogenates from *Socs4^R108X/R108X^* and wild-type mice infected with X31 virus. In general, we did not observe significant differences in cytokine and chemokine levels in the latter phase of the infection (days 5–7; data not shown). However, at day 3 post-infection, the *Socs4^R108X/R108X^* mice showed significantly higher levels of cytokines and chemokines, such as IL-1β, IL-4, IL-5, IL-6, IL-12p40, IL-13, IFN-γ, and KC (CXCL1), MIP-2 (CXCL2) and MCP-1 (CCL2), respectively (Fig. 2A). There were elevated but not significantly different, levels of TGF-β and IL-10, and no difference was detected in levels of several other cytokines and chemokines (data not shown). The elevated levels of pro-inflammatory cytokines, in particular IL-6, IFNγ and IL-1β, are likely to account for the increased morbidity observed in the *Socs4* mutant mice. No difference was observed between *Socs4^R108X/R108X^* and control mice in cytokine and chemokine production in either spleen or lungs on d3 following systemic administration of polyinosinic-polycytidylic acid (poly I:C), a non-dynamic, virus-like stimulus (Fig. S2). This indicates that the increased cytokine/chemokine production (Fig. 2A) is a specific response to infection with influenza virus, and may reflect the modestly increased viral titres on d3 (Fig. 1E). It further suggests that that the Toll-like receptor (TLR)3 pathway is not perturbed and therefore not directly regulated by SOCS4. This is consistent with our data suggesting a hematopoietic, rather than an innate epithelial defect.

In order to determine whether the elevated cytokine levels in the lungs were caused by increased production per infiltrating cell or increased numbers of a particular subset of cells, we profiled infected lungs on day 3 post-infection. While total cell numbers in *Socs4^R108X/R108X^*-infected lungs were slightly elevated compared to wild-type controls, this difference did not reach statistical significance (Fig. 2B, top panel). Similarly, we observed slightly elevated levels of B cells and NK cells in *Socs4^R108X/R108X^*-infected lungs and no difference in neutrophil or macrophage numbers, including alveolar macrophages (CD11c^+^ Ly6C^int^ F4/80^+^) and inflammatory monocytes (CD11c^-^ Ly6G^low^ Ly6C^+^ F4/80^+^) (Fig. 2B, middle panels). Surprisingly, modest but significantly higher levels of total T cells (TCRβ^+^) including higher levels of CD4 (CD44^+^) and CD8 (CD44^+^) T cells were detected in the lungs on day 3 post-infection (Fig. 2B, bottom panels). No difference was observed on day 2 post-infection, whereas on day 6 the cellularity of lungs of wild-type controls was higher than in SOCS4 mutant mice (Fig. S3). We also confirmed *Socs4* expression in the infiltrating immune cells recovered by bronchioalveolar lavage (BAL). *Socs4* mRNA expression increased over time, peaking at day 3 post-infection (due either to the changing cellular composition or to up-regulation within the infiltrating cells). In contrast, *Socs4* mRNA was ∼15-fold lower in equivalent amounts of total lung RNA (Fig. 2C).

Together, these data suggest that activated T cells (not necessarily virus-specific, but CD44^hi^) accumulate in the lungs during the initial phase of infection in response to the elevated cytokine and chemokine levels. This may be a response to elevated chemoattractant levels or may potentially result from cytokine-driven proliferation (a bystander reaction rather than antigen-driven expansion). In summary, there is an increased net production of pro-inflammatory cytokines and chemokines, and while the initiating defect remains unclear, SOCS4 appears to have a classical role as a negative regulator of cytokine production and/or response in the innate immune reaction to influenza infection.

### *Socs4^R108X/R108X^* mice display altered tissue distribution of influenza-specific CD8 T cells

**Figure 3:**
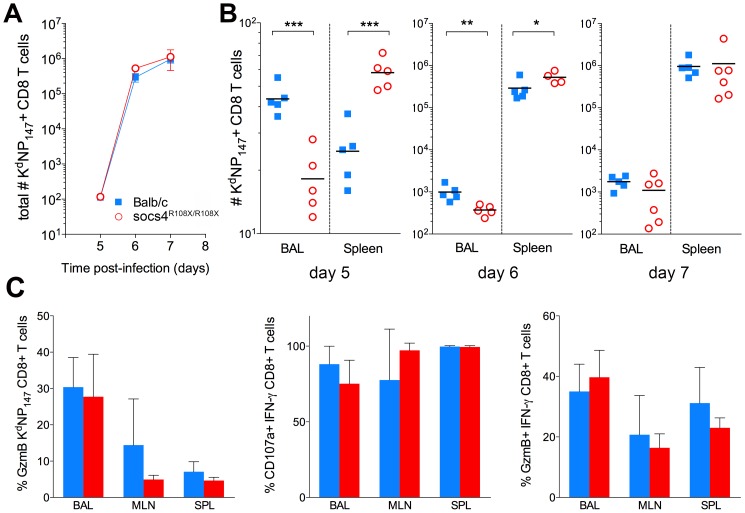
Altered tissue distribution of virus-specific CD8 T cells in *Socs4^R108X/R108X^* mice. (A) Total numbers (BAL+MLN+SPL) of virus-specific (K^d^NP_147_ positive) CD8 T cells following X31 influenza virus infection. Mean values (n = 5) are plotted, error bars represent SEM. (B) Significant differences were observed in the distribution of K^d^NP_147_ positive CD8 T cells in lungs (BAL) and spleens of *Socs4^R108X/R108X^* and Balb/c control mice following infection. *p<0.05, **<0.005, ***<0.001 (C) Percentage of GzmB positive K^d^NP_147_ positive CD8 T cells in various tissues on d10 post-infection (left hand panel); percentage of CD107a positive (middle panel) or GzmB positive (right hand panel) IFN-γ positive CD8 T cells on d10 post-infection following *ex vivo* stimulation with K^d^NP_147_ peptide. Mean values (n = 5) are plotted, error bars represent SD.

Following intranasal inoculation with virus, CD8 T cells are primed and activated in the mediastinal lymph node (MLN) [29] and the anti-viral competence of these cells then depends on their ability to migrate to the site of infection [30]. We therefore used MHC class I tetramer staining to track and quantify influenza-specific (K^d^NP_147_) CD8 T cells in MLN, BAL and spleen, at days 5, 6 and 7 post-infection. Overall, the expansion of K^d^NP_147_-CD8 T cells did not differ between *Socs4^R108X/R108X^* mice and wild-type controls (Fig. 3A). However, at days 5 and 6 post-infection *Socs4^R108X/R108X^* mice had significantly less virus-specific cells at the site of infection (BAL) when compared to controls and instead, these cells appeared to localise to the spleen. No difference was observed on day 7 post-infection (Fig. 3B), at which time-point, viral titers in the majority of *Socs4^R108X/R108X^* mice were below detection levels (Fig. 1E). No differences were observed in MLN during the course of experiment (data not shown).

Although, virus-specific cells did not traffic efficiently to the site of infection, the clearance of virus on day 7 post-infection indicates that the loss of SOCS4 does not affect the ability of CD8 T cells to kill the virus. To confirm this, CTLs were analysed for markers of cytotoxic degranulation and function. Mice were primed with X31 and cells were harvested from lungs, draining lymph nodes and spleen (day 10 post-infection). K^d^NP_147_-CD8 T cells were analysed for granzyme B (GzmB) production as well as IFN-γ, TNF and IL-2. Alternatively, cells were stimulated *ex vivo* with K^d^NP_147_ peptide and stained with antibodies against lysosome-associated membrane protein 1 (LAMP-1, CD107a). No differences in GzmB production, cytokine production or CD107a mobilisation were observed between *Socs4^R108X/R108X^* mice and wild-type controls (Fig. 3C; data not shown).

### *Socs4* mutant T cells exhibit defects in activation

**Figure 4:**
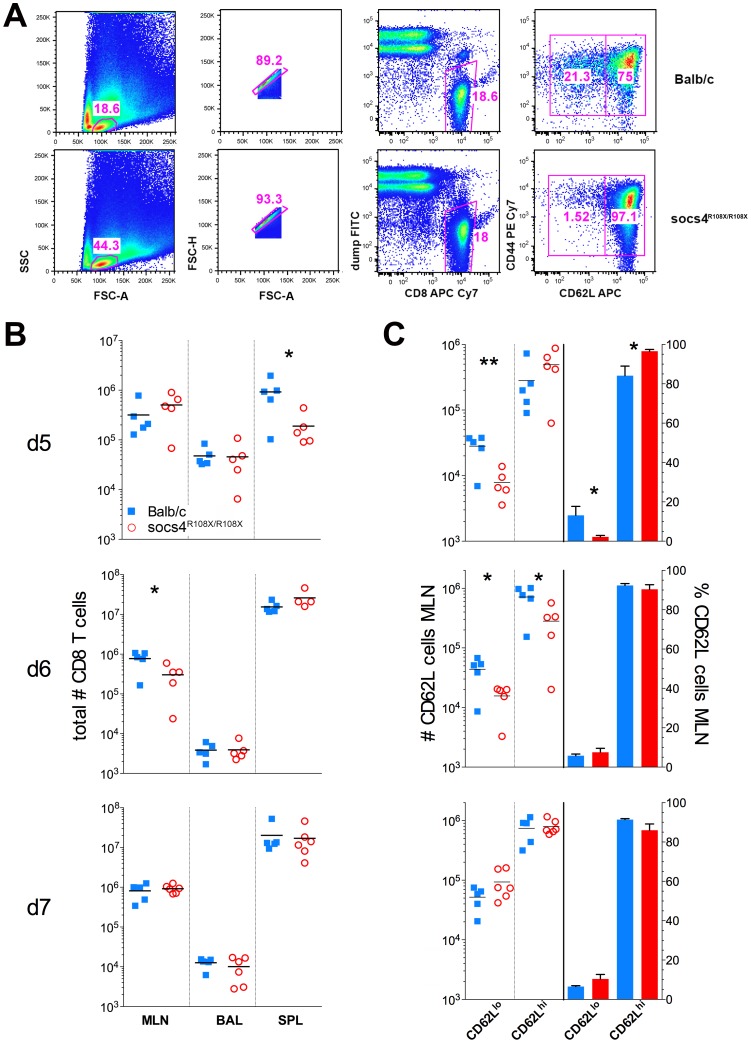
Reduced downregulation of CD62L expression on *Socs4^R108X/R108X^* CD8 cells in following X31 influenza infection. (A) Flow cytometric analysis showing the gating strategy and percentages of CD62L^hi^ and CD62L^l^°CD8 T cells on day 5 post-infection with X31 influenza virus. Representative dot plots are shown from control Balb/c and *Socs4^R108X/R108X^* mice. (B) Total CD8 T cell numbers in the lymph node (MLN), lungs (BAL) and spleen (SPL) of *Socs4^R108X/R108X^* and Balb/C mice on day d5, d6 and d7 following infection with X31 influenza virus. (C) Total number and percentages of CD62L^hi^ and CD62L^l^°CD8 cells in the MLN of *Socs4^R108X/R108X^* and Balb/C mice following X31 influenza virus infection.

The defect in trafficking of K^d^NP_147_-CD8 cells to the site of infection could result from reduced activation of the T cells following MHC-antigen presentation or alternatively, from altered homing signals. To investigate this further, we examined a panel of T cell activation markers (CD62L, CD69, CD44, CD25) and homing receptors (CCR5, CXCR3, CCR7) at days 5, 6 and 7 post-infection. The majority of markers examined did not differ between wild-type and *Socs4^R108X/R108X^* CD8 T cells (data not shown). However, at day 5 post-infection *Socs4^R108X/R108X^* CD8 cells in the draining lymph nodes (MLN) showed comparatively higher levels of CD62L expression (Fig. 4), reflecting reduced activation. By day 6 there were reduced numbers of CD62L positive *Socs4^R108X/R108X^* CD8 cells in the lymph nodes (MLN), with no differences in the percentage of CD62L^hi^ versus CD62L^lo^ expressing cells (Fig. 4B and C), and no differences observed at day 7 post-infection (Fig. 4B and C).

We also reconstituted *Socs4^R108X/R108X^* mice with wild-type Thy1.1 donor bone marrow. When analysed 8 weeks post-reconstitution for expression of congenic markers, we observed a much lower degree of chimerism (50–84% in CD4 and 43–78% in CD8 compartments) (Fig. S4A), compared to reconstitution of wild-type mice with *Socs4^R108X/R108X^* or wild-type donor cells. This result suggests that SOCS4 may regulate stromal signals, and although unlikely to contribute to the defective anti-viral response described here, warrants further independent investigation. Nevertheless, this competitive reconstitution enabled us to investigate the contribution of both donor and recipient cells to anti-influenza responses. Consistent with the level of chimerism, a higher proportion of wild-type donor cells were detected in the spleens and MLNs of all *Socs4^R108X/R108X^* recipient mice at day 6 post-infection. In contrast, the ratios of donor and recipient CD4 and CD8 T cells in the lungs (BAL) were approximately equal (Fig. S4B). Interestingly, the *Socs4^R108X/R108X^* Thy1.2 T cells in the lungs appeared to be significantly less activated than wild-type Thy1.1 cells, as evidenced by lower CD69 levels (Fig. S4C), whilst all T cells showed similar levels of CD44 on their surface (Fig. S4D).

Together, these data point to influenza-specific T cells receiving inadequate and/or incorrect signals in the lymph nodes, which in the *Socs4^R108X/R108X^* mice, results in their inability to migrate to the site of infection. In turn, this results in a reduced ability to clear the virus. To investigate this further, CD4 and CD8 T cells were isolated from *Socs4^R108X/R108X^* and wild-type mice, and signaling responses analysed following CD3 engagement.

### SOCS4 regulates signaling downstream of TCR stimulation

**Figure 5:**
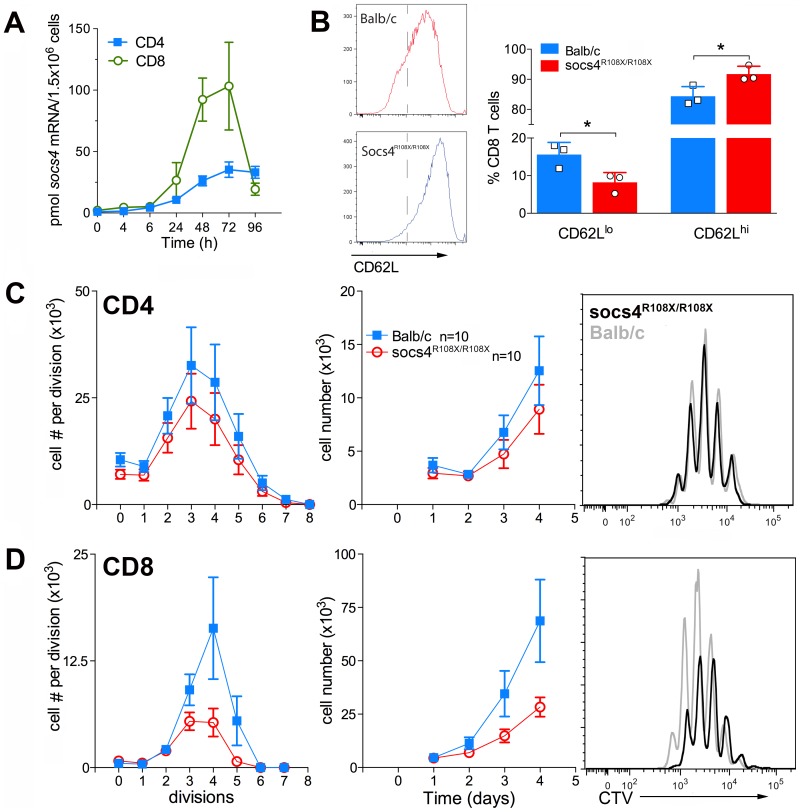
Defective TCR responses in *Socs4^R108X/R108X^* CD8 T cells. CD4 or CD8 T cells were purified from *Socs4^R108X/R108X^* or wild-type spleens by negative selection, plated onto anti-CD3 coated plates in the presence of IL-2 and analysed at the indicated timepoints. (A) Q-PCR analysis showing increased expression of *Socs4* mRNA in purified T cells from Balb/c mice following TCR engagement (mean±SEM, n = 3). (B) Cells were analysed by flow cytometry for the expression of the T cell activation marker, CD62L (mean±S.D.), n = cells derived from 3 mice. (C–D) Cells were labelled with Cell Trace Violet (CTV) for analysis of proliferative responses. Cell numbers per division are shown in the left hand panels; total cell numbers in the centre panels, (C) CD4 T cells, (D) CD8 T cells. Results are shown as the mean±S.E.M. using pooled data from 2 independent experiments (n = 5 mice per group per experiment). Representative histograms are plotted for each subset (day 4; right hand panels).

We initially examined SOCS4 expression in purified splenic CD4 and CD8 cells following activation with anti-CD3 antibodies. SOCS4 expression was compatible with a role in TCR signaling, with *Socs4* mRNA induced within 24 h of TCR stimulation with anti-CD3 antibodies, and peaking at 72 h. In addition, the magnitude of *Socs4* expression was 4-fold higher in CD8 versus CD4 cells (Fig. 5A). To investigate T cell responses in the absence of functional SOCS4, purified CD8 T cells from *Socs4^R108X/R108X^* or wild-type mice were stimulated with anti-CD3 antibodies and analysed by flow cytometry for the expression of the T cell activation markers CD69, CD44, CD25 and CD62L. In comparison to wild-type cells, surface expression of CD62L remained high, indicating that the *Socs4* mutant cells responded poorly to TCR activation (Fig. 5B). CD69, CD44 and CD25 expression levels were comparable in wild-type and *Socs4^R108X/R108X^* cells (data not shown). To further investigate the consequences of reduced TCR responses, cells were labelled with Cell Trace Violet (CTV) dye, and proliferation measured in response to stimulation with anti-CD3 antibodies. As shown in Fig. 5C and D, anti-CD3-induced proliferation was impaired in *Socs4^R108X/R108X^* CD8, but not in CD4, T cells. The defective proliferation seemed to result from a decrease in the number of cells that were mobilizing per division (Fig.5C, left panel) and resulted in a corresponding decrease in the total number of cells by day 4 of culture (Fig. 5D, middle panel). These differences resulted from a proliferative defect as opposed to differences in the rate of cell death, since the percentage of propidium iodide positive cells was equivalent for both *Socs4^R108X/R108X^* and wild-type cells (data not shown).

These results indicate a defect that is intrinsic to the *Socs4^R108X/R108X^* T cells and further suggest that signaling through the TCR may be qualitatively different, thus accounting for the reduced activation observed in the lymph nodes during infection and *in vitro* following TCR engagement.

## Discussion

Loss of functional SOCS4 protein led to a dramatic phenotype following influenza A infection. *Socs4^R108X/R108X^* mice were highly susceptible to primary infection with the virulent PR8 H1N1 strain exhibiting increased mortality associated with weight loss and delayed viral clearance. Similar results were observed following infection with the less virulent X31 H3N2 strain, however the anorexia was somewhat ameliorated, enabling us to dissect the underlying pathology in greater detail. The transient weight loss induced by influenza infection is known to reflect viral pathogenicity and as in this study, correlates with increased cytokine/chemokine levels in the lungs [2]. This is the first description of SOCS4-deficient mice and suggests that SOCS4 will play an important role in immune regulation during infection. Deletion of the C-terminal 328 amino acids removes the main functional domains, the SH2 domain and SOCS box, and the conserved SOCS4/5 N-terminal motif [7] is no longer intact. There is no described biological function for the remaining 108 amino acids of SOCS4. The long N-terminal regions of SOCS proteins are predicted to be largely disordered [7], and although such regions within a full-length molecule can play a role in multi-protein complex formation, it is unlikely that a short, disordered fragment will be functional. We have however, been able to express the truncated 108-residue fragment in 293T cells (data not shown). It is possible that, if expressed *in vivo*, the 108-residue fragment could retain binding to its endogenous target and compete off other signaling intermediates, acting as a dominant negative. It might be speculated that residues 1–108 are involved in binding to a receptor subunit in a similar fashion to SOCS5 binding to the IL-4Rα [31]but the identity of such a receptor complex is currently unknown. Regardless, given that expression of this putative fragment is under the control of the SOCS4 promoter, the observed phenotype indicates that normal SOCS4 function has been disrupted at the endogenous level and reflects a biological role for SOCS4 in regulating anti-viral immunity to influenza A.

So far very little is known regarding the role of different SOCS proteins in influenza infection, outside of the ability of SOCS1 and SOCS3 to regulate specific cytokine receptor complexes. SOCS1 and SOCS3 have been implicated as negative regulators of innate immune responses (type I interferons) via a RIG-I dependent pathway [32], [33], [34]. SOCS3, but not SOCS1, has also been shown to inhibit type I interferon signaling via an NF-κB-dependent pathway [35]. SOCS1 and SOCS3 expression has been associated with symptomatic influenza infection, whereas SOCS2 and SOCS5 have been linked to asymptomatic disease [36].

The pathogenicity of virulent influenza infections is not well understood, but it is accepted that pathogenic strains (such as PR8) cause dysregulation of innate immunity resulting in aberrant cytokine and chemokine production (also known as a cytokine storm), which results in the lung tissue damage. Following infection with a viral strain of relatively moderate virulence, the absence of a functional SOCS4 protein led to dysregulated cytokine production akin to a cytokine storm. This uncontrolled inflammatory response is most likely responsible for the excessive weight loss in *Socs4^R108X/R108X^* mice, but it is unclear whether this increased cytokine production also contributes to the delayed viral clearance. Given that the delay in clearance is most pronounced during the adaptive phase of the immune response we postulate that the two phenomena (weight loss and delayed viral clearance) are independent of each other and result from multiple defects caused by the absence of a functional SOCS4.

Airway epithelial cells are the primary targets for influenza infection and are capable of producing cytokines as early as 3–6 hours post-infection [37]. Similarly, a variety of immunomodulators are produced by infected monocytes and macrophages [38]. IL-6 and IL-1β were among the proinflammatory cytokines elevated in the lungs of *Socs4^R108X/R108X^* mice (Fig. 2A). IL-6 is known to promote pulmonary inflammation and is a potential biomarker for patients at risk following infection with H1N1 [39]. In addition to its anti-viral activity [40], IL-1 has also been shown to contribute to acute inflammatory lung pathology following influenza infection [41]. The effects of IL-1 and IL-6 are synergistic [42] with both hyper-induced in H5N1 infected human macrophages leading to acute respiratory distress [43]. Although IL-6 expression is dysregulated in *Socs4^R108X/R108X^* mice, it does not follow that SOCS4 regulates IL-6 directly. We have tested this theory (data not shown) and are confident that SOCS4 does not directly inhibit gp130 signaling. The elevated levels of MCP-1 and IL-12p40 are also of interest. IL-12p40 is produced by epithelial cells [44] and forms a homodimer (IL-12p80) with both monomeric and dimeric forms implicated in instigating the inflammatory response in lungs [45]. High MCP-1 levels have been associated with both the profound inflammatory responses observed in *Ifitm3^-/-^* mice infected with influenza [46], and with the increased severity of disease observed in humans with a variant *Ifitm3* gene [47].

Surprisingly, we did not detect major differences in the inflammatory cell infiltrates in the lungs of *Socs4^R108X/R108X^* and control mice. Numbers of neutrophils, NK cells, macrophages and inflammatory monocytes were comparable between the two groups. We do not understand the mechanism by which the lack of functional SOCS4 protein results in enhanced cytokine/chemokine production, but given the negligible expression of *Socs4* in lung tissue and the relatively equal number of infiltrating cells, we propose that multiple immune cell types are producing greater amounts of cytokine per cell. This is also consistent with retention of the weight loss phenotype in wild-type mice reconstituted with *Socs4^R108X/R108X^* bone marrow.

However, our analysis of infected lungs did reveal significantly elevated numbers of T cells on day 3 post-infection, with both CD4 and CD8 showing an activated CD44^hi^ phenotype. This increased number of T cells in the lungs of *Socs4^R108X/R108X^* mice is suggestive of proliferation due to bystander activation driven by cytokines rather than antigen-driven expansion of specific T cells or stimulation of cell division by cross-reactive antigens [48], [49], [50]. Interestingly, IL-6 appears to be one of the major factors driving the spontaneous proliferation of naïve T cells (both CD8 and CD4) [51], [52], [53]. Geginat et al. [52] further demonstrated that IL-4 in combination with IL-6 (and other cytokines such as IL-10, IL-12 and TNF) selectively induced the proliferation of naïve CD4 T cells. In our *Socs4^R108X/R108X^* mice we observed significantly increased levels of IL-4 and IL-6 as well as elevated levels of IL-10, which combined with other cytokines in a complex lung milieu might lead to the activation and proliferation of T cells already present in the lungs [54].

In addition to the early excessive inflammation, we observed a clear difference in the distribution of virus-specific CD8 T cells in *Socs4^R108X/R108X^* mice, with tetramer positive cells appearing to accumulate in the spleen of *Socs4^R108X/R108X^* mice instead of migrating to the site of infection, and this discrepancy was particularly apparent by day 5 post-infection (Fig. 3B). Activation of CD8 T cells occurs in response to TCR engagement by antigen in the draining lymph nodes. CD62L expression on naïve T cells is downregulated, with concomitant upregulation of the surface markers CD44, CD25 and CD69 [55]. The most highly activated T cells are then thought to traffic to the lungs, whereas less activated T cells migrate to the spleen and peripheral LNs [29]. Thus, cells migrating to the spleen display a CD69 low/negative phenotype, whilst CD8 T cells in the lungs show a CD69 high phenotype [29]. Consistent with this concept, CD62L down-regulation was less pronounced on *Socs4^R108X/R108X^* CD8 cells in the draining lymph nodes at day 5, suggesting that despite the high levels of virus, these cells were not being activated to the same extent as wild-type cells. On day 6 post-infection, *Socs4^R108X/R108X^* (Thy1.2) CD8 T cells migrating to the lungs had significantly lower levels of CD69 expression than wild-type (Thy1.1) CD8 cells. Although both subsets appear to be recruited to the lungs in similar ratios, their activation status differed significantly in an identical environment. Given that this result is in the context of a chimeric bone marrow reconstitution where up to 60% of the hematopoietic cells were wild-type, it strongly indicates an intrinsic CD8 T cell defect. CD69 expression declines in the absence of antigenic stimulation [56], however, again the high viral load in the lungs indicates that adequate levels of antigen were available for presentation in the lymph nodes, so that even in the presence of antigenic stimulation *Socs4^R108X/R108X^* T cells showed a much weaker activation compared to the wild-type cells.

We conclude that this defect in activation and trafficking of *Socs4^R108X/R108X^* CD8 cells to the lungs is likely to be linked to the reduced activation of the virus-specific cells, which in turn suggests impaired TCR activation and signaling. The significantly lower numbers of influenza-specific effector cells available in the *Socs4^R108X/R108X^* lungs no doubt result in the much higher viral loads observed (Fig. 1).

A defect in TCR signaling is supported by our *in vitro* data showing differences in the activation marker CD62L and the reduced capacity of *Socs4^R108X/R108X^* CD8 T cells to proliferate in response to anti-CD3-activation. Similar proliferative deficiencies have been observed previously in various settings where TCR signaling was affected [57], [58]. The *in vitro* proliferative defect (Fig. 5C) is in contrast to the normal expansion of CD8 T cells in response to influenza A infection, as indicated by total numbers of virus-specific CD8 T cells (Fig. 3A). However, such a discrepancy might be due to differences in CD3 versus peptide/MHC stimulation and/or several factors present *in vivo* that are not accounted for in the *in vitro* setting, including cytokine milieu or co-receptor stimulation [57]. In fact, *in vitro* co-stimulation alone can overcome a proliferative defect in cells stimulated only with anti-CD3 antibodies [59]. Therefore, *in vivo* viral challenge might be sufficient to induce a normal level of T cell expansion and overcome a proliferative impairment detected *in vitro*. CD62L is an L-selectin that plays a major role in directing lymphocytes to the site of infection and inflammation. Downregulation of CD62L reflects the activation status of T cells and is linked to gene transcription, mRNA stability and shedding from the surface due to increased activity of membrane proteases[60]. We suggest that loss of SOCS4 may affect TCR signalling resulting in maintained CD62L expression. We have also identified differences in CD69 expression between *Socs4^R108X/R108X^* and control CD8 T cells supporting this conclusion and indicating that other activation markers can also be affected, depending on the experimental context. We currently postulate that SOCS4 regulates TCR signalling rather than CD62L shedding, although at this stage we cannot rule out the involvement of SOCS4 in ubiquitylation of membrane proteases involved in CD62L shedding.

Our data describe for the first time a phenotype for *Socs4^R108X/R108X^* mice in the context of a viral challenge. While SOCS4 plays a negative regulatory role in inflammatory responses to influenza, it appears to be a positive regulator of TCR signaling. The former is consistent with the traditional role of SOCS proteins and a plausible mechanism of action is through inhibition of the JAK/STAT pathways, although the exact target/s are currently unknown. The positive involvement in TCR signaling is a novel function for a SOCS protein.

## Materials and Methods

### Ethics statement

All mice were bred at the Walter and Eliza Hall Institute's animal facility. Animal experiments followed the NHMRC Code of Practice for the Care and Use of Animals for Scientific Purposes guidelines and were approved by the Walter and Eliza Hall Institute's Animal Ethics Committee (AEC 2008.032 and 2011.031).

### Animals

SOCS4 mutant mice (*Socs4^R108X/R108X^*) were generated through ENU mutagenesis by Ingenium Pharmaceuticals AG (Martinsried, Germany) on the C3HeB/FeJ background. The *Socs4^R108X^* mutation was subsequently backcrossed onto both the Balb/c and C57BL/6 backgrounds for 10 generations.

### Virus infection

Mice were lightly anaesthetized by inhalation of methoxyflurane, and infected intranasally (i.n.) with 20 plaque forming units (pfu) of PR8 (A/Puerto Rico/8/1934 (H1N1)) or 10^3^-10^4^ pfu of X31 (A/X31(H3N2)) influenza virus in 30 µL PBS. Virus stocks were grown in the allantoic cavity of 10 day old embryonated chicken eggs, from which the viral titre was determined by plaque assay on monolayers of Madin derby canine kidney (MDCK) cells. The weight of mice was monitored daily from day 3 post-infection. Mice were euthanised at various time points following infection and tissues collected for analysis.

### Determination of viral titres

Lungs taken from mice after primary viral infection were homogenised and the virus-containing supernatant above the cell debris was harvested and stored at −80°C. Titres of infectious virus in the lung supernatants were determined by plaque assay on monolayers of MDCK cells.

### Tissue sampling and cell preparation

Spleen, mediastinal lymph node (MLN), and bronchoalveolar lavage (BAL) samples were recovered from mice at different stages during the acute phase of the primary infection. BAL samples were incubated on plastic petri-dishes for 1 h at 37°C to remove macrophages for tetramer experiments or used for phenotypic staining without adherent cell depletion. BAL fluid was collected and stored at −80°C for cytokine analysis. The spleens and MLN were disrupted, processed to single-cell suspensions and enriched for CD8 T cells by negative depletion using goat anti-mouse IgG and IgM antibodies to non-CD8 cell lineages (Jackson ImmunoResearch Labs, West Grove, PA, USA). Lungs collected from infected mice were minced, incubated in 2 mg/mL collagenase for 30 min at 37°C, and processed to a single-cell suspension.

### Tetramer and phenotypic staining

Cells from BAL, MLN and spleen were stained with a tetramer conjugated to Strepavidin-PE (Molecular Probes, Eugene, OR, USA) at an optimal staining dilution (1∶100 K^d^NP_147_) for 1 h at room temperature (RT). All batches of tetramer used in this study were titrated and the optimal dilution was based on both the percentage of epitope-specific CD8 T cells and the mean fluorescence intensity (MFI) of tetramer staining. Cells were washed twice in FACS buffer, and stained with antibodies to CD4, B220 and F4/80 (all FITC conjugated, dump gate), CD8-APC Cy7, CD3ε-PerCP Cy5.5, CD62L-APC, CD44-PE Cy7 (BD Biosciences or BioLegend) for 30 min on ice, washed twice and analysed by flow cytometry on the FACS Canto (BD Biosciences) and analysed by FlowJo software (Tree Star). For phenotypic staining, different combinations of antibodies were used as indicated in the text.

### Granzyme B and CD107a staining

Cells stained for tetramer and CD8α were fixed and permeabilized using a BD Cytofix/Cytoperm kit (BD Pharmingen), then stained for intracellular GzmB using anti-human GzmB-APC (clone GB12; Caltag Laboratories) as previously described [61]. Acquisition of cell surface CD107a was used to measure activation-induced degranulation by antigen-specific CD8^+^ T cells [62]. Briefly, cells were incubated for 1 h at 37°C with 1 µM NP_147–155_ peptide, 10 U/ml hrIL-2 and FITC-conjugated anti-CD107a (clone 1D4B; BD Pharmingen). Monensin (1 µl/ml; BD Pharmingen) was then added and cells were incubated for a further 4 h. Cells were stained for surface CD8α expression, then fixed and permeabilized before intracellular staining with anti-IFN-γ FITC (clone XMG1.2; BioLegend) and anti-GzmB APC. Negative controls incubated in the absence of peptide were used to control for spontaneous production of cytokine or expression of CD107a.

### Bone marrow chimeras

To establish chimeras, recipient mice (Balb/c Thy1.1) were irradiated with two doses of 550R 3 h apart. At 3–5 h after the final irradiation, recipients were reconstituted with 3×10^6^ T cell-depleted bone marrow (BM) cells isolated from femurs and tibias of donor mice (Thy1.2 *Socs4^R108X/R108X^* or Balb/c). Briefly, bone marrow was flushed from the femurs of 5–7 week old mice, washed once and incubated in complement-fixing antibodies anti-CD4 (RL172), anti-CD8 (3.186) and anti-Thy-1 (J1j) for 30 min on ice. After washing the cells once in HEPES Earle's medium containing 2.5% fetal calf serum (FCS) (HEM2.5), antibody-binding cells were incubated in rabbit complement at 37°C for 20 min. The mice were allowed to reconstitute for at least 8 weeks prior to use, blood samples were collected (by retro-orbital bleeding), and reconstitution of T cell compartment assessed by FACS analysis.

### Cytokine analyses

Cytokine levels were assessed by capture ELISA or BioPlex Pro Assay (BioRad, CA, USA). Lung homogenates were stored at −80°C prior to analysis. BioPlex detection was performed according to the manufacturer's instructions. For ELISAs, U-bottom 96-well plates (Costar, NY, USA) were coated overnight at 4°C with capture antibodies against IL-4, IL-6, IL-10, IFN-γ, GM-CSF, KC, MIP-1α or MIP-2 (BD Pharmingen, CA or RD Systems, MN, USA) and were then incubated with serial dilutions of samples for 2 h at RT, washed and incubated with the appropriate biotinylated detection antibody for 2 h at RT, followed by incubation with streptavidin-horseradish peroxidase. Plates were developed with 3,3′,5,5′-tetramethylbenzidine (TMB) in 0.1 M sodium acetate pH 6, and colour development terminated by the addition of 2N H_2_SO_4_. Absorbance values were read at 450 nm. TGF-β ELISA was performed according to the manufacturer's instructions (RD Systems, MN, USA).

### *In vitro* T cell proliferation assay

Single-cell suspensions were generated from spleens, and CD8 or CD4 T cells purified by negative selection using magnetic beads (Dynabeads, Invitrogen or BioMag, Qiagen). Enrichment of cells was verified by flow cytometry. Purified T cells were labelled with Cell Trace Violet (CTV) (Molecular Probes, OR, USA) according to the manufacturer's instructions. *In vitro* stimulation assays were performed by plating cells at 10^4^ cells per well in RPMI 1640 medium containing 10% (v/v) heat-inactivated FCS (Sigma-Aldrich, MO, USA), 5×10^−5^ M 2-ME (Sigma-Aldrich), 100 µg/mL streptomycin and 100 U/mL penicillin (Invitrogen Life Technologies) into anti-CD3-coated (10 µg/mL, clone KT3-1-1) 96-well plates. Recombinant mouse IL-2 (20 ng/mL) was also added to wells. Cells were harvested at different timepoints, propidium iodide (2 µg/mL) and 5000 of Sphero Nile Red Fluorescent Particles (BD Biosciences) added per well and cell division analysed by flow cytometry. Each sample was analysed in duplicate.

### Real-time quantitative PCR (Q-PCR)

Q-PCR analysis of *Socs4* mRNA expression was performed essentially as described [63].

### Statistical analyses

Statistical analyses were performed using the unpaired t-test provided within GraphPad Prism 5 software and Compare Groups of Growth Curves software package available on the Walter and Eliza Hall Institute Bioinformatics Division's website http://bioinf.wehi.edu.au/.

## Supporting Information

Figure S1 ***Socs4^R108X/R108X^*** **mice show normal composition of peripheral immune cells and normal thymic development.** (A) Peripheral blood differential counts of *Socs4^R108X/R108X^* and Balb/c mice. White Blood Cell differential count (left graph) and Complete Blood Counts for red and white blood cells (middle graph) and platelets (right graph) were performed using an ADVIA 2120 Haematology Analyser. Mean values from 18 *Socs4^R108X/R108X^* mice and 10 Balb/c mice are plotted; error bars represent S.D. (B) Normal thymic development in *Socs4^R108X/R108X^* mice. Thymocytes were stained with anti-CD4, CD8, CD44 and CD25 antibodies and DN (double negative CD4/CD8^-^) subdivided based on their CD44 and CD25 profile (DN1 CD44+CD25-, DN2 CD44+ CD25+, DN3 CD44-CD25+ and DN4 CD44-CD25-) as well as CD4 and CD8 expression. Mean values (n = 5) were plotted, error bars represent S.D. (C) Percentage of T and B cells in various naïve tissues of *Socs4^R108X/R108X^* and Balb/c mice. Cells were gated as double positive T cells, either TCRβ+CD4+ or TCRβ+CD8+, or as B220+ B cells. Mean values are plotted (n = 5); error bars represent S.D.
(TIF)

Figure S2 **Cytokine and chemokine production in spleen and lungs of** ***Socs4^R108X/R108X^*** **following administration of polyinosinic-polycytidylic acid.** *Socs4^R108X/R108X^* and control mice were injected intraperitoneally with 2 µg of poly I:C per g of body weight. Lungs and spleens were harvested on day 3 post-injection and cytokine and chemokine levels in tissue homogenates were analysed by Bioplex. Mean ± S.E.M. are shown for biological replicates (n = 5 for Balb/c, n = 5 for *Socs4^R108X/R108X^*).
(TIF)

Figure S3 **Phenotypic analysis of lung hematopoietic subsets in** ***Socs4^R108X/R108X^*** **and Balb/c mice at day 2 (A) and day 6 (B) post-infection.** Flow cytometry analysis was performed on homogenized lungs. * indicates p<0.05, **<0.005.
(TIF)

Figure S4 ***Socs4^R108X/R108X^*** **mice reconstituted with wild-type bone marrow show reduced engraftment and impaired activation of Thy1.2 T cells following H3N2 influenza A infection.** (A) Irradiated *Socs4^R108X/R108X^* mice were reconstituted with wild-type Thy1.1 bone marrow and expression of congenic markers (Thy1.1, Thy1.2) analysed in the T cell compartment in peripheral blood at 8 weeks post-reconstitution. (B–F) Lethally irradiated *Socs4^R108X/R108X^* Thy1.2 mice reconstituted with wild-type Thy1.1 bone marrow were infected with H3N2 X31 influenza A virus. Spleen (SPL), mediastinal lymph node (LN) and BAL were collected on day 6 post-infection and single cell suspensions were analysed by flow cytometry to examine (B) percentage of Thy1.1 vs Thy1.2 cells, (C) CD69 and (D) CD44 expression on Thy1.1 and Th1.2 CD4 and CD8 T cells. Mean±S.D. are shown from biological replicates (n = 6), ** p = 0.001–0.01, ***p = 0.0001–0.001, ****p<0.0001. MFI, Mean Fluorescence Index. *Socs4^R108X/R108X^* mice reconstituted with wild-type bone marrow (WT > *Socs4^R108X/R108X^*) show no difference in disease severity when compared to wild-type (Thy1.2) mice reconstituted with wild-type (Thy1.1) bone marrow (WT(Thy1.1)>WT(Thy1.2)). Mice were infected with H3N2 X31 influenza A virus and weight loss (E) was monitored for 6 days, at which timepoint lungs were harvested for viral titre estimation (F). No statistically significant differences were observed between the groups.
(TIF)

## References

[1] Newall AT, Scuttham PA, Hodgkinson B (2007) Economic Report into the cost of influenza to the Australian Health System. http://www.influenzaspecialistgrouporgau/images/stories/docs/isg_cost_influenza_report_30_2007pdf.

[2] La Gruta NL, Kedzierska K, Stambas J, Doherty PC (2007) A question of self-preservation: immunopathology in influenza virus infection. Immunol Cell Biol 85: 85–92. 17213831 10.1038/sj.icb.7100026

[3] Arankalle VA, Lole KS, Arya RP, Tripathy AS, Ramdasi AY, et al (2010) Role of host immune response and viral load in the differential outcome of pandemic H1N1 (2009) influenza virus infection in Indian patients. PLoS One 5: e13099. 20957032 10.1371/journal.pone.0013099 PMC2948498

[4] Thomas P, Keating R, Hulse-Post D, Doherty P (2006) Cell-mediated protection in influenza infection. Emerg Infect Dis 12: 48–54. 16494717 10.3201/eid1201.051237 PMC3291410

[5] Alexander WS (2002) Suppressors of cytokine signalling (SOCS) in the immune system. Nat Rev Immunol 2: 410–416. 12093007 10.1038/nri818

[6] Hilton DJ, Richardson RT, Alexander WS, Viney EM, Willson TA, et al (1998) Twenty proteins containing a C-terminal SOCS box form five structural classes. Proc Natl Acad Sci U S A 95: 114–119. 9419338 10.1073/pnas.95.1.114 PMC18144

[7] Feng ZP, Chandrashekaran IR, Low A, Speed TP, Nicholson SE, et al (2012) The N-terminal domains of SOCS proteins: a conserved region in the disordered N-termini of SOCS4 and 5. Proteins 80: 946–957. 22423360 10.1002/prot.23252

[8] Zhang JG, Farley A, Nicholson SE, Willson TA, Zugaro LM, et al (1999) The conserved SOCS box motif in suppressors of cytokine signaling binds to elongins B and C and may couple bound proteins to proteasomal degradation. Proc Natl Acad Sci U S A 96: 2071–2076. 10051596 10.1073/pnas.96.5.2071 PMC26738

[9] Linossi EM, Nicholson SE (2012) The SOCS box-adapting proteins for ubiquitination and proteasomal degradation. IUBMB Life 64: 316–323. 22362562 10.1002/iub.1011

[10] Kershaw NJ, Murphy JM, Liau NP, Varghese LN, Laktyushin A, et al (2013) SOCS3 binds specific receptor-JAK complexes to control cytokine signaling by direct kinase inhibition. Nat Struct Mol Biol 20: 469–476. 23454976 10.1038/nsmb.2519 PMC3618588

[11] Babon JJ, Kershaw NJ, Murphy JM, Varghese LN, Laktyushin A, et al (2012) Suppression of cytokine signaling by SOCS3: characterization of the mode of inhibition and the basis of its specificity. Immunity 36: 239–250. 22342841 10.1016/j.immuni.2011.12.015 PMC3299805

[12] Greenhalgh CJ, Rico-Bautista E, Lorentzon M, Thaus AL, Morgan PO, et al (2005) SOCS2 negatively regulates growth hormone action in vitro and in vivo. J Clin Invest 115: 397–406. 15690087 10.1172/JCI22710 PMC546423

[13] Endo T, Sasaki A, Minoguchi M, Joo A, Yoshimura A (2003) CIS1 interacts with the Y532 of the prolactin receptor and suppresses prolactin-dependent STAT5 activation. J Biochem 133: 109–113. 12761205 10.1093/jb/mvg004

[14] Lavens D, Montoye T, Piessevaux J, Zabeau L, Vandekerckhove J, et al (2006) A complex interaction pattern of CIS and SOCS2 with the leptin receptor. J Cell Sci 119: 2214–2224. 16684815 10.1242/jcs.02947

[15] Alexander WS, Starr R, Fenner JE, Scott CL, Handman E, et al (1999) SOCS1 is a critical inhibitor of interferon gamma signaling and prevents the potentially fatal neonatal actions of this cytokine. Cell 98: 597–608. 10490099 10.1016/s0092-8674(00)80047-1

[16] Metcalf D, Greenhalgh CJ, Viney E, Willson TA, Starr R, et al (2000) Gigantism in mice lacking suppressor of cytokine signalling-2. Nature 405: 1069–1073. 10890450 10.1038/35016611

[17] Croker BA, Metcalf D, Robb L, Wei W, Mifsud S, et al (2004) SOCS3 is a critical physiological negative regulator of G-CSF signaling and emergency granulopoiesis. Immunity 20: 153–165. 14975238 10.1016/s1074-7613(04)00022-6

[18] Croker BA, Krebs DL, Zhang JG, Wormald S, Willson TA, et al (2003) SOCS3 negatively regulates IL-6 signaling in vivo. Nat Immunol 4: 540–545. 12754505 10.1038/ni931

[19] Kario E, Marmor MD, Adamsky K, Citri A, Amit I, et al (2005) Suppressors of cytokine signaling 4 and 5 regulate epidermal growth factor receptor signaling. J Biol Chem 280: 7038–7048. 15590694 10.1074/jbc.M408575200

[20] Bullock AN, Rodriguez MC, Debreczeni JE, Songyang Z, Knapp S (2007) Structure of the SOCS4-ElonginB/C complex reveals a distinct SOCS box interface and the molecular basis for SOCS-dependent EGFR degradation. Structure 15: 1493–1504. 17997974 10.1016/j.str.2007.09.016 PMC2225448

[21] Hu G, Zhou R, Liu J, Gong AY, Chen XM (2010) MicroRNA-98 and let-7 regulate expression of suppressor of cytokine signaling 4 in biliary epithelial cells in response to Cryptosporidium parvum infection. J Infect Dis 202: 125–135. 20486857 10.1086/653212 PMC2880649

[22] Sasi W, Jiang WG, Sharma A, Mokbel K (2010) Higher expression levels of SOCS 1,3,4,7 are associated with earlier tumour stage and better clinical outcome in human breast cancer. BMC Cancer 10: 178. 20433750 10.1186/1471-2407-10-178 PMC2876081

[23] Sutherland JM, Keightley RA, Nixon B, Roman SD, Robker RL, et al (2012) Suppressor of cytokine signaling 4 (SOCS4): moderator of ovarian primordial follicle activation. J Cell Physiol 227: 1188–1198. 21604262 10.1002/jcp.22837

[24] Augustin M, Sedlmeier R, Peters T, Huffstadt U, Kochmann E, et al (2005) Efficient and fast targeted production of murine models based on ENU mutagenesis. Mamm Genome 16: 405–413. 16075367 10.1007/s00335-004-3028-2

[25] Bender BS, Croghan T, Zhang L, Small PA Jr (1992) Transgenic mice lacking class I major histocompatibility complex-restricted T cells have delayed viral clearance and increased mortality after influenza virus challenge. J Exp Med 175: 1143–1145. 1552285 10.1084/jem.175.4.1143 PMC2119177

[26] Denton AE, Doherty PC, Turner SJ, La Gruta NL (2007) IL-18, but not IL-12, is required for optimal cytokine production by influenza virus-specific CD8+ T cells. Eur J Immunol 37: 368–375. 17219365 10.1002/eji.200636766

[27] Imai Y, Kuba K, Neely GG, Yaghubian-Malhami R, Perkmann T, et al (2008) Identification of oxidative stress and Toll-like receptor 4 signaling as a key pathway of acute lung injury. Cell 133: 235–249. 18423196 10.1016/j.cell.2008.02.043 PMC7112336

[28] Peiris JS, Hui KP, Yen HL (2010) Host response to influenza virus: protection versus immunopathology. Curr Opin Immunol 22: 475–481. 20594815 10.1016/j.coi.2010.06.003 PMC2927395

[29] Lawrence CW, Braciale TJ (2004) Activation, differentiation, and migration of naive virus-specific CD8+ T cells during pulmonary influenza virus infection. J Immunol 173: 1209–1218. 15240712 10.4049/jimmunol.173.2.1209

[30] Lawrence CW, Ream RM, Braciale TJ (2005) Frequency, specificity, and sites of expansion of CD8+ T cells during primary pulmonary influenza virus infection. J Immunol 174: 5332–5340. 15843530 10.4049/jimmunol.174.9.5332

[31] Seki Y, Hayashi K, Matsumoto A, Seki N, Tsukada J, et al (2002) Expression of the suppressor of cytokine signaling-5 (SOCS5) negatively regulates IL-4-dependent STAT6 activation and Th2 differentiation. Proc Natl Acad Sci U S A 99: 13003–13008. 12242343 10.1073/pnas.202477099 PMC130576

[32] Pothlichet J, Chignard M, Si-Tahar M (2008) Cutting edge: innate immune response triggered by influenza A virus is negatively regulated by SOCS1 and SOCS3 through a RIG-I/IFNAR1-dependent pathway. J Immunol 180: 2034–2038. 18250407 10.4049/jimmunol.180.4.2034

[33] Wei H, Wang S, Chen Q, Chen Y, Chi X, et al (2014) Suppression of Interferon Lambda Signaling by SOCS-1 Results in Their Excessive Production during Influenza Virus Infection. PLoS Pathog 10: e1003845. 24391501 10.1371/journal.ppat.1003845 PMC3879354

[34] Ramirez-Martinez G, Cruz-Lagunas A, Jimenez-Alvarez L, Espinosa E, Ortiz-Quintero B, et al (2013) Seasonal and pandemic influenza H1N1 viruses induce differential expression of SOCS-1 and RIG-I genes and cytokine/chemokine production in macrophages. Cytokine 62: 151–159. 23434273 10.1016/j.cyto.2013.01.018 PMC4148900

[35] Pauli EK, Schmolke M, Wolff T, Viemann D, Roth J, et al (2008) Influenza A virus inhibits type I IFN signaling via NF-kappaB-dependent induction of SOCS-3 expression. PLoS Pathog 4: e1000196. 18989459 10.1371/journal.ppat.1000196 PMC2572141

[36] Huang Y, Zaas AK, Rao A, Dobigeon N, Woolf PJ, et al (2011) Temporal dynamics of host molecular responses differentiate symptomatic and asymptomatic influenza A infection. PLoS Genet 7: e1002234. 21901105 10.1371/journal.pgen.1002234 PMC3161909

[37] Chan MC, Cheung CY, Chui WH, Tsao SW, Nicholls JM, et al (2005) Proinflammatory cytokine responses induced by influenza A (H5N1) viruses in primary human alveolar and bronchial epithelial cells. Respir Res 6: 135. 16283933 10.1186/1465-9921-6-135 PMC1318487

[38] Julkunen I, Sareneva T, Pirhonen J, Ronni T, Melen K, et al (2001) Molecular pathogenesis of influenza A virus infection and virus-induced regulation of cytokine gene expression. Cytokine Growth Factor Rev 12: 171–180. 11325600 10.1016/s1359-6101(00)00026-5

[39] Paquette SG, Banner D, Zhao Z, Fang Y, Huang SS, et al (2012) Interleukin-6 is a potential biomarker for severe pandemic H1N1 influenza A infection. PLoS One 7: e38214. 22679491 10.1371/journal.pone.0038214 PMC3367995

[40] Janeway C Jr, Medzhitov R (2000) Viral interference with IL-1 and toll signaling. Proc Natl Acad Sci U S A 97: 10682–10683. 11005852 10.1073/pnas.97.20.10682 PMC34044

[41] Schmitz N, Kurrer M, Bachmann MF, Kopf M (2005) Interleukin-1 is responsible for acute lung immunopathology but increases survival of respiratory influenza virus infection. J Virol 79: 6441–6448. 15858027 10.1128/JVI.79.10.6441-6448.2005 PMC1091664

[42] Le JM, Fredrickson G, Reis LF, Diamantstein T, Hirano T, et al (1988) Interleukin 2-dependent and interleukin 2-independent pathways of regulation of thymocyte function by interleukin 6. Proc Natl Acad Sci U S A 85: 8643–8647. 3263651 10.1073/pnas.85.22.8643 PMC282515

[43] Cheung CY, Poon LL, Lau AS, Luk W, Lau YL, et al (2002) Induction of proinflammatory cytokines in human macrophages by influenza A (H5N1) viruses: a mechanism for the unusual severity of human disease? Lancet 360: 1831–1837. 12480361 10.1016/s0140-6736(02)11772-7

[44] Mikols CL, Yan L, Norris JY, Russell TD, Khalifah AP, et al (2006) IL-12 p80 is an innate epithelial cell effector that mediates chronic allograft dysfunction. Am J Respir Crit Care Med 174: 461–470. 16728708 10.1164/rccm.200512-1886OC PMC2648123

[45] Cooper AM, Khader SA (2007) IL-12p40: an inherently agonistic cytokine. Trends Immunol 28: 33–38. 17126601 10.1016/j.it.2006.11.002

[46] Everitt AR, Clare S, Pertel T, John SP, Wash RS, et al (2012) IFITM3 restricts the morbidity and mortality associated with influenza. Nature 484: 519–523. 22446628 10.1038/nature10921 PMC3648786

[47] Zhang YH, Zhao Y, Li N, Peng YC, Giannoulatou E, et al (2013) Interferon-induced transmembrane protein-3 genetic variant rs12252-C is associated with severe influenza in Chinese individuals. Nat Commun 4: 1418. 23361009 10.1038/ncomms2433 PMC3562464

[48] Murali-Krishna K, Altman JD, Suresh M, Sourdive DJ, Zajac AJ, et al (1998) Counting antigen-specific CD8 T cells: a reevaluation of bystander activation during viral infection. Immunity 8: 177–187. 9491999 10.1016/s1074-7613(00)80470-7

[49] Tough DF, Borrow P, Sprent J (1996) Induction of bystander T cell proliferation by viruses and type I interferon in vivo. Science 272: 1947–1950. 8658169 10.1126/science.272.5270.1947

[50] Di Genova G, Savelyeva N, Suchacki A, Thirdborough SM, Stevenson FK (2010) Bystander stimulation of activated CD4+ T cells of unrelated specificity following a booster vaccination with tetanus toxoid. Eur J Immunol 40: 976–985. 20104490 10.1002/eji.200940017

[51] Tajima M, Wakita D, Noguchi D, Chamoto K, Yue Z, et al (2008) IL-6-dependent spontaneous proliferation is required for the induction of colitogenic IL-17-producing CD8+ T cells. J Exp Med 205: 1019–1027. 18426983 10.1084/jem.20071133 PMC2373835

[52] Geginat J, Sallusto F, Lanzavecchia A (2001) Cytokine-driven proliferation and differentiation of human naive, central memory, and effector memory CD4(+) T cells. J Exp Med 194: 1711–1719. 11748273 10.1084/jem.194.12.1711 PMC2193568

[53] Unutmaz D, Pileri P, Abrignani S (1994) Antigen-independent activation of naive and memory resting T cells by a cytokine combination. J Exp Med 180: 1159–1164. 8064232 10.1084/jem.180.3.1159 PMC2191658

[54] Cose S, Brammer C, Khanna KM, Masopust D, Lefrancois L (2006) Evidence that a significant number of naive T cells enter non-lymphoid organs as part of a normal migratory pathway. Eur J Immunol 36: 1423–1433. 16708400 10.1002/eji.200535539

[55] Marzio R, Mauel J, Betz-Corradin S (1999) CD69 and regulation of the immune function. Immunopharmacol Immunotoxicol 21: 565–582. 10466080 10.3109/08923979909007126

[56] Testi R, D'Ambrosio D, De Maria R, Santoni A (1994) The CD69 receptor: a multipurpose cell-surface trigger for hematopoietic cells. Immunol Today 15: 479–483. 7945773 10.1016/0167-5699(94)90193-7

[57] Smith-Garvin JE, Burns JC, Gohil M, Zou T, Kim JS, et al (2010) T-cell receptor signals direct the composition and function of the memory CD8+ T-cell pool. Blood 116: 5548–5559. 20847203 10.1182/blood-2010-06-292748 PMC3031403

[58] Han J, Shui JW, Zhang X, Zheng B, Han S, et al (2005) HIP-55 is important for T-cell proliferation, cytokine production, and immune responses. Mol Cell Biol 25: 6869–6878. 16055701 10.1128/MCB.25.16.6869-6878.2005 PMC1190228

[59] D'Souza WN, Chang CF, Fischer AM, Li M, Hedrick SM (2008) The Erk2 MAPK regulates CD8 T cell proliferation and survival. J Immunol 181: 7617–7629. 19017950 10.4049/jimmunol.181.11.7617 PMC2847891

[60] Rainer TH (2002) L-selectin in health and disease. Resuscitation 52: 127–141. 11841880 10.1016/s0300-9572(01)00444-0

[61] Jenkins MR, Kedzierska K, Doherty PC, Turner SJ (2007) Heterogeneity of effector phenotype for acute phase and memory influenza A virus-specific CTL. J Immunol 179: 64–70. 17579022 10.4049/jimmunol.179.1.64

[62] Betts MR, Brenchley JM, Price DA, De Rosa SC, Douek DC, et al (2003) Sensitive and viable identification of antigen-specific CD8+ T cells by a flow cytometric assay for degranulation. J Immunol Methods 281: 65–78. 14580882 10.1016/s0022-1759(03)00265-5

[63] Lee C, Kolesnik TB, Caminschi I, Chakravorty A, Carter W, et al (2009) Suppressor of cytokine signalling 1 (SOCS1) is a physiological regulator of the asthma response. Clin Exp Allergy 39: 897–907. 19309352 10.1111/j.1365-2222.2009.03217.x PMC3449009

