# Supplementary figures and images for: Suppressor of Cytokine Signaling 4 (SOCS4) Protects against Severe Cytokine Storm and Enhances Viral Clearance during Influenza Infection
Source: PLoS Pathog. 2014 May 8;10(5):e1004134. doi: 10.1371/journal.ppat.1004134 (PMC4014316; doi:10.1371/journal.ppat.1004134)

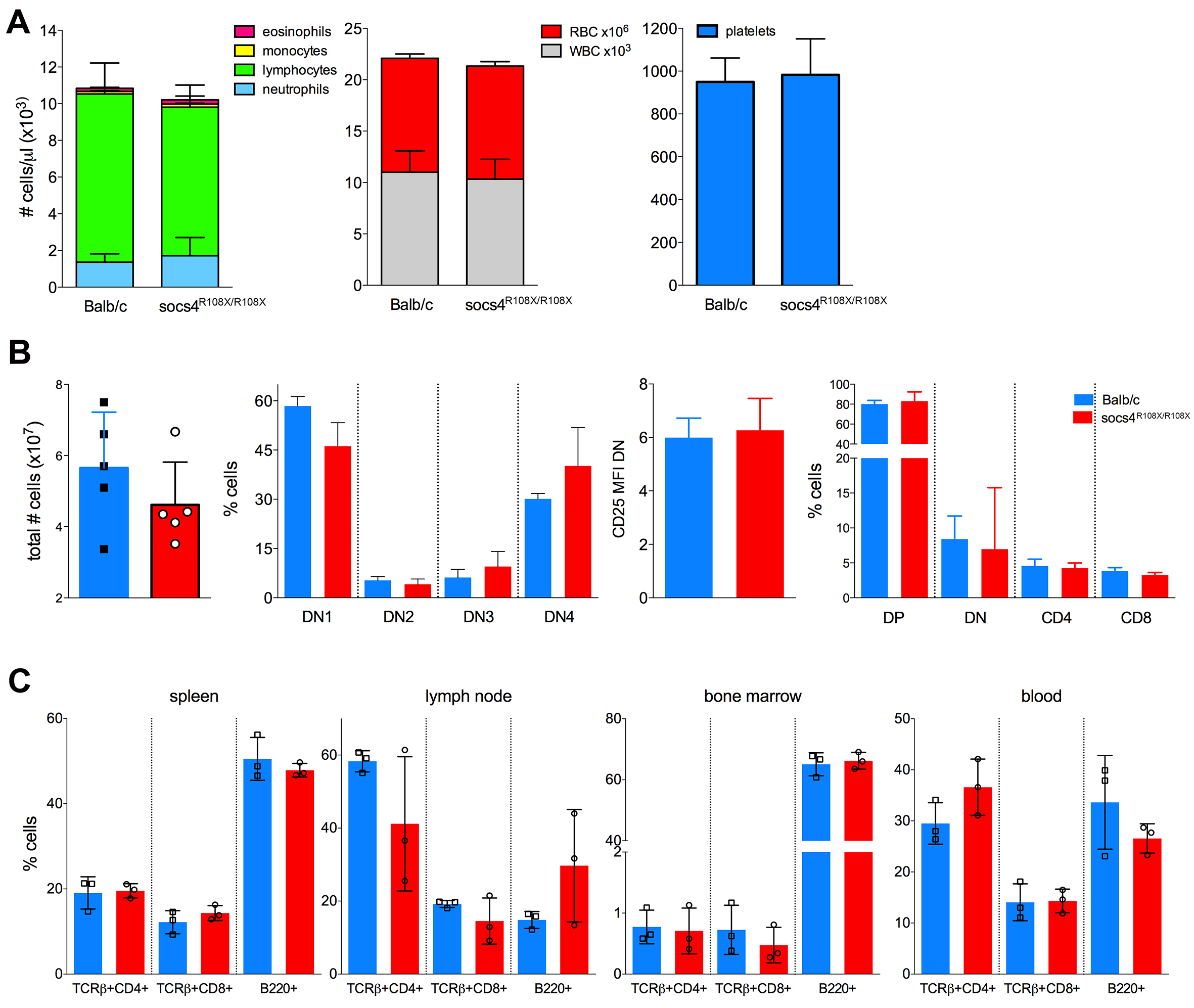

Supplement: Figure S1 — Socs4R108X/R108X mice show normal composition of peripheral immune cells and normal thymic development. (A) Peripheral blood differential counts of Socs4R108X/R108X and Balb/c mice. White Blood Cell differential count (left graph) and Complete Blood Counts for red and white blood cells (middle graph) and platelets (right graph) were performed using an ADVIA 2120 Haematology Analyser. Mean values from 18 Socs4R108X/R108X mice and 10 Balb/c mice are plotted; error bars represent S.D. (B) Normal thymic development in Socs4R108X/R108X mice. Thymocytes were stained with anti-CD4, CD8, CD44 and CD25 antibodies and DN (double negative CD4/CD8-) subdivided based on their CD44 and CD25 profile (DN1 CD44+CD25-, DN2 CD44+ CD25+, DN3 CD44-CD25+ and DN4 CD44-CD25-) as well as CD4 and CD8 expression. Mean values (n = 5) were plotted, error bars represent S.D. (C) Percentage of T and B cells in various naïve tissues of Socs4R108X/R108X and Balb/c mice. Cells were gated as double positive T cells, either TCRβ+CD4+ or TCRβ+CD8+, or as B220+ B cells. Mean values are plotted (n = 5); error bars represent S.D. (TIF) [file ppat.1004134.s001.tif]

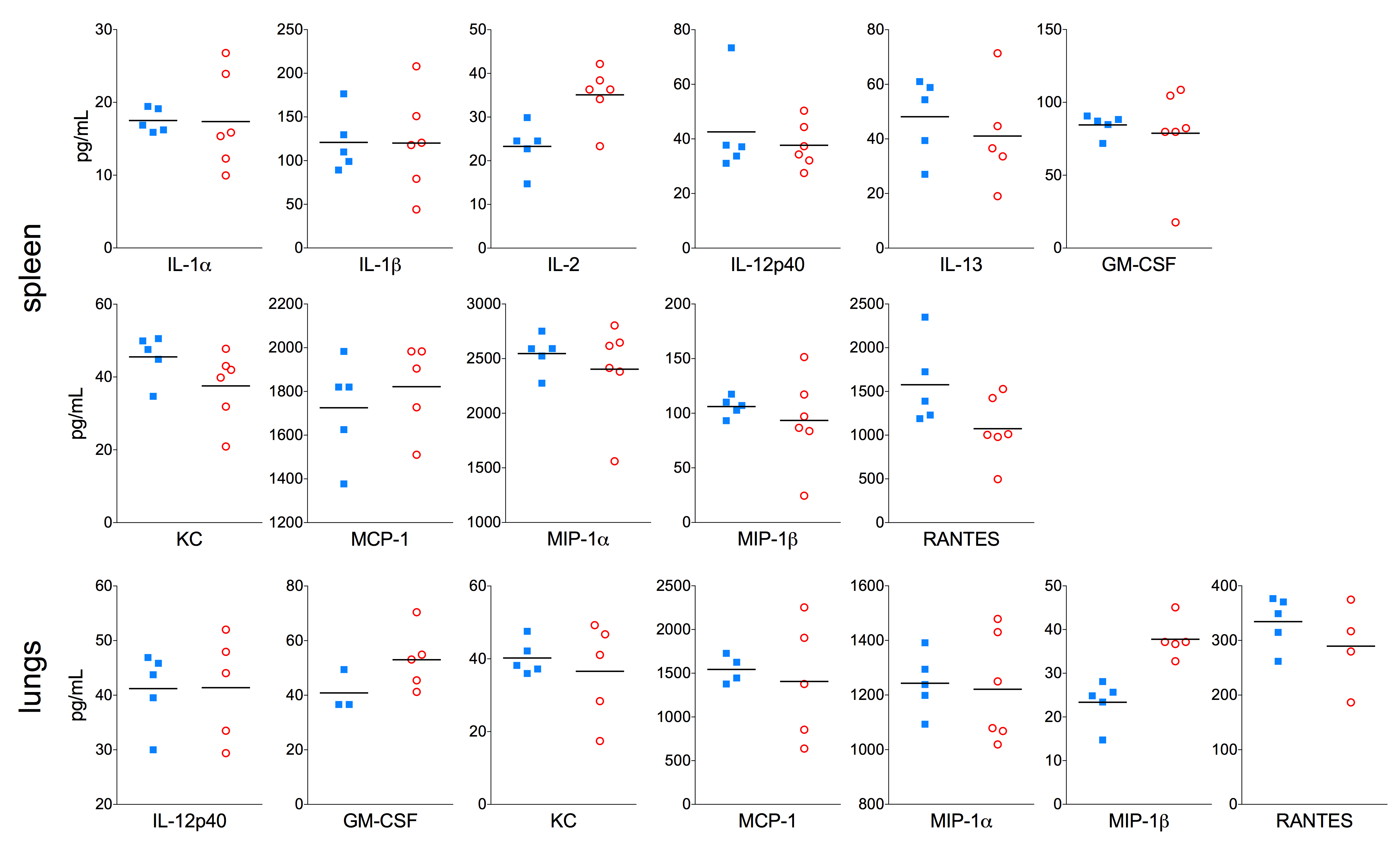

Supplement: Figure S2 — Cytokine and chemokine production in spleen and lungs of Socs4R108X/R108X following administration of polyinosinic-polycytidylic acid. Socs4R108X/R108X and control mice were injected intraperitoneally with 2 µg of poly I:C per g of body weight. Lungs and spleens were harvested on day 3 post-injection and cytokine and chemokine levels in tissue homogenates were analysed by Bioplex. Mean ± S.E.M. are shown for biological replicates (n = 5 for Balb/c, n = 5 for Socs4R108X/R108X). (TIF) [file ppat.1004134.s002.tif]

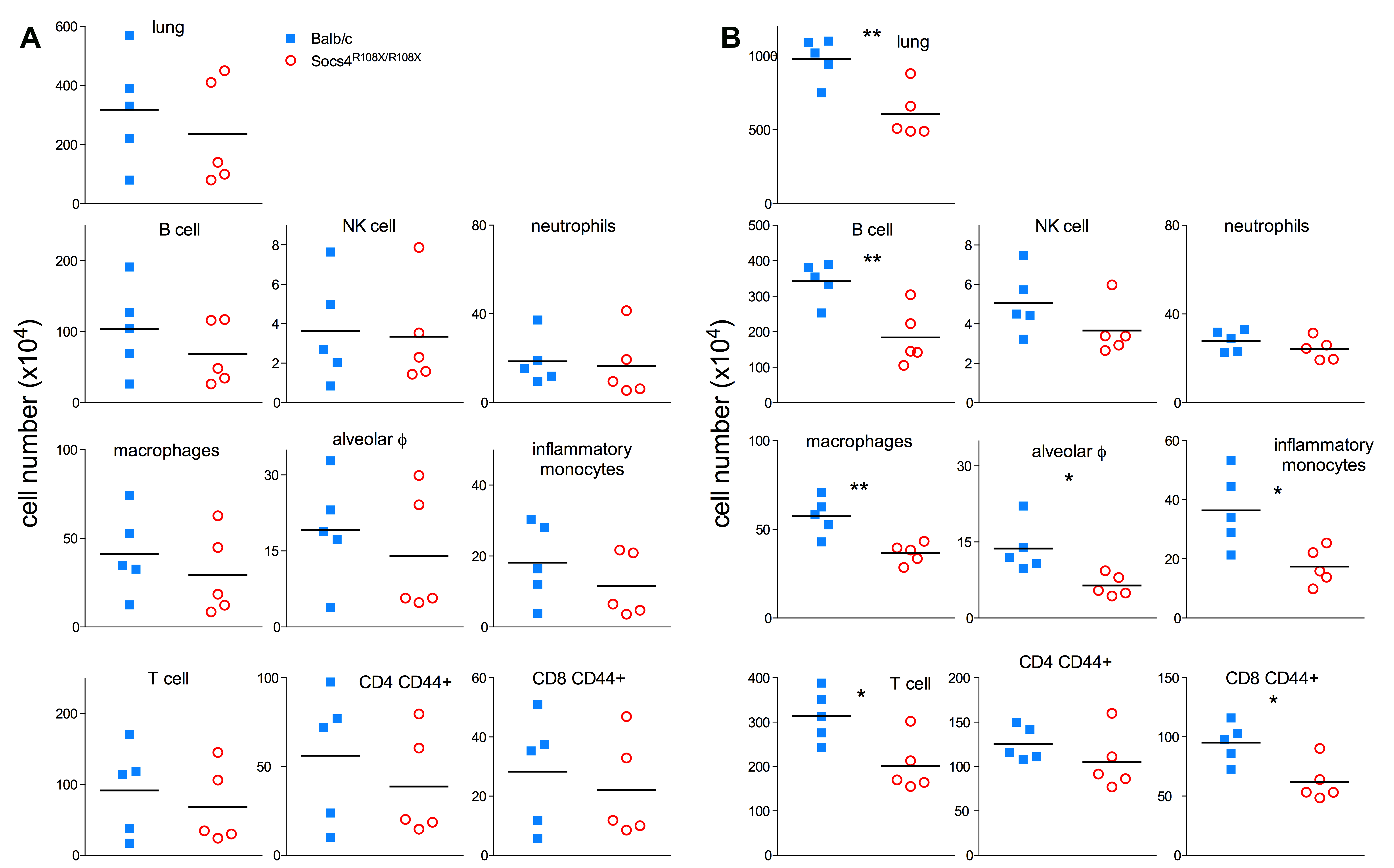

Supplement: Figure S3 — Phenotypic analysis of lung hematopoietic subsets in Socs4R108X/R108X and Balb/c mice at day 2 (A) and day 6 (B) post-infection. Flow cytometry analysis was performed on homogenized lungs. * indicates p<0.05, **<0.005. (TIF) [file ppat.1004134.s003.tif]

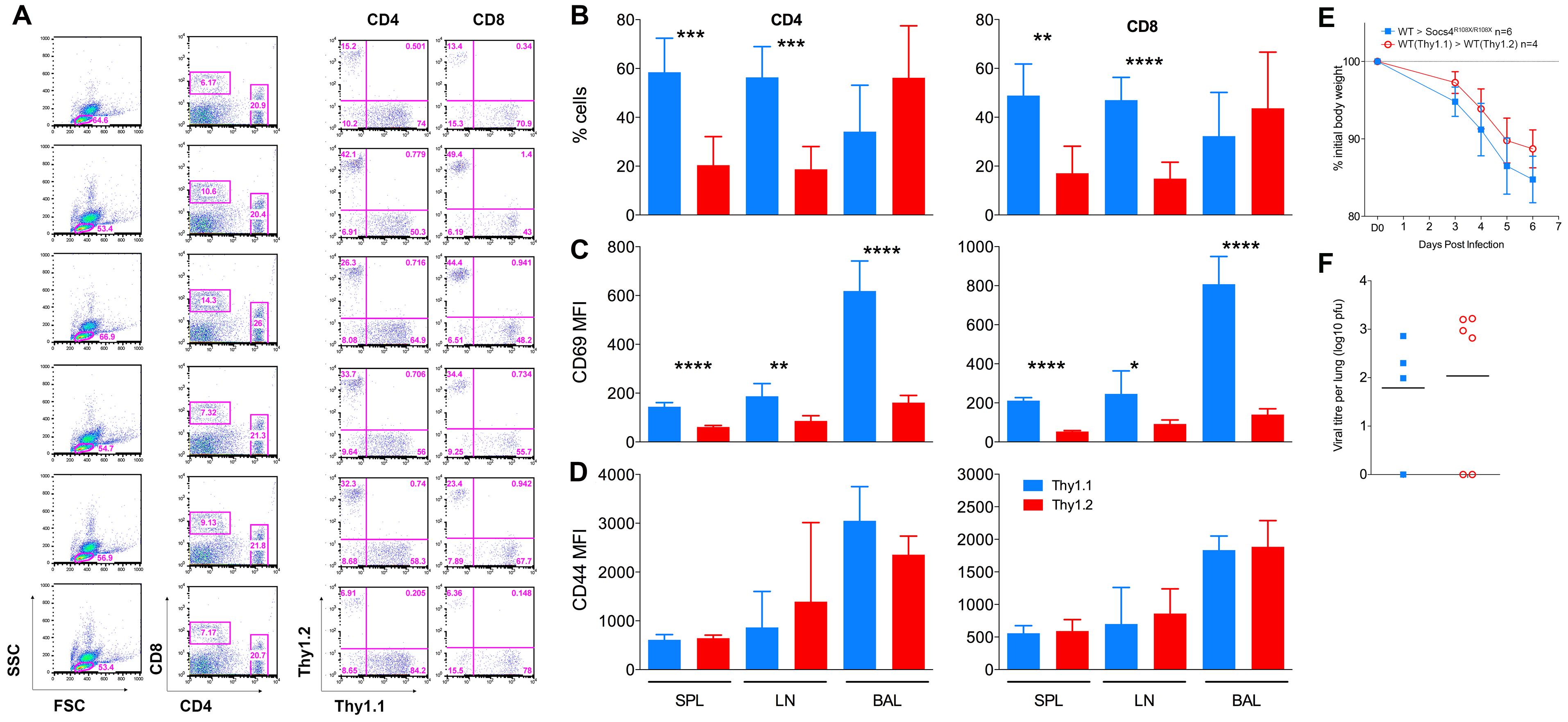

Supplement: Figure S4 — Socs4R108X/R108X mice reconstituted with wild-type bone marrow show reduced engraftment and impaired activation of Thy1.2 T cells following H3N2 influenza A infection. (A) Irradiated Socs4R108X/R108X mice were reconstituted with wild-type Thy1.1 bone marrow and expression of congenic markers (Thy1.1, Thy1.2) analysed in the T cell compartment in peripheral blood at 8 weeks post-reconstitution. (B–F) Lethally irradiated Socs4R108X/R108X Thy1.2 mice reconstituted with wild-type Thy1.1 bone marrow were infected with H3N2 X31 influenza A virus. Spleen (SPL), mediastinal lymph node (LN) and BAL were collected on day 6 post-infection and single cell suspensions were analysed by flow cytometry to examine (B) percentage of Thy1.1 vs Thy1.2 cells, (C) CD69 and (D) CD44 expression on Thy1.1 and Th1.2 CD4 and CD8 T cells. Mean±S.D. are shown from biological replicates (n = 6), ** p = 0.001–0.01, ***p = 0.0001–0.001, ****p<0.0001. MFI, Mean Fluorescence Index. Socs4R108X/R108X mice reconstituted with wild-type bone marrow (WT > Socs4R108X/R108X) show no difference in disease severity when compared to wild-type (Thy1.2) mice reconstituted with wild-type (Thy1.1) bone marrow (WT(Thy1.1)>WT(Thy1.2)). Mice were infected with H3N2 X31 influenza A virus and weight loss (E) was monitored for 6 days, at which timepoint lungs were harvested for viral titre estimation (F). No statistically significant differences were observed between the groups. (TIF) [file ppat.1004134.s004.tif]
